# Reviews and Book Notices

**Published:** 1877-08

**Authors:** 


### Reviews and Book Notices.

The Microscopist: A Manual of Microscopy and Compendium of the Microscopic Sciences, Micro-Mineralogy, Micro-Chemistry, Biology, Histology, and Pathological Histology. Third edition, re-written and greatly enlarged, with two hundred and five illustrations. By *J. H. Wythe, A. M., M. D.*, Professor of Microscopy and Biology in the Medical College of the Pacific, San Francisco. Philadelphia: *Lindsay & Blakiston*, 1877.

The reappearance of this book, one of the oldest, if not the oldest work on the subject which has been published in the United States, is an indication of the interest which microscopical studies have attracted for the last twenty-five years in this country, and would lead one to expect a high character of a book which has kept its popularity so long.

The book, exclusive of the preface and index, containing 253 pages, is divided into thirteen chapters. Chapter I. is made up of remarks upon the history of the microscope and its uses. Chapter II. deals with the theory and construction of simple and compound microscopes. Chapter III. gives a sketch of some of the accessories of the instrument, and chapter IV. deals with the use of the instrument and test objects. Chapter V. on methods of examining objects, and chapter VI. on mounting and preserving objects. These six chapters occupy sixty-seven pages of matter, and bring us to page 84.

Here the author leaves the general consideration of the subject of microscopy and passes on to particular lines of study. He gives us one chapter (VII.) on the use of the microscope in geology and mineralogy; one (VIII.) on the use of the microscope in chemistry; one (IX.) on the cell theory. Chapter X. gives a sketch of the lower orders of vegetable life, beginning with the vegetable cell and its parts, the vessels, and other structures found in plants, and then the various substances, as starch, gum, oils, etc., of which they are composed. He then gives a description of the lower orders of plants, including fungi, diatoms, etc. Chapter XI. is made up of a list of the lower forms of animal life; chapter XII. gives an outline of human histology, and chapter XIII. is taken up with the use of the microscope in pathological investigations.

It will be seen that the work includes a wide range of topics, the shortest of which if treated with completeness would more than fill a volume the size of the present one. It may be worth while to go over this book with some minuteness, and see how our author has succeeded in his difficult task of putting an encyclopædia in such a short compass.

Chapter II. is a condensation of the brief and puerile sketch of optics usually found in the ordinary works on the microscope. It seems to us that it is time for such chapters to be expunged from works upon the practical use of the microscope. They are an attempt to popularize the science of optics, a thing as easy to do as to popularize mathematics or Greek, the attempt only ends in filling valuable space with material which is useless at the best, and generally teaches positive error. Those who wish to learn the optical principles of the microscope should go to works on optics.

Our author, instead of improving upon the usual character of such chapters, has rather fallen below it. We need only refer to an example or two. On page 25, (fig. 3.) he give as an illustration of the construction of an acromatic object-glass, a form that was obsolete fifteen years ago. On page 26, he says that the ordinary Huygenian eye-piece is composed of two plane convex lenses, with their plane sides next the eye, *whose focal lengths are as* 3 *to* 1, and which are placed a distance apart equal to half the sum of their focal lengths.

This is, to be sure, an old formula, but is not used so far as we are aware, by any modern maker. The focal lengths of the glasses are more nearly 2 to 1. Merz, of Munich, uses exactly that proportion; his 1½ eye-piece has lenses of 2 and 1 inch focal lengths placed about two inches apart. Beck's A eye-piece has lenses of 2½ and 1⅝ inches focal length. Scarcely any two makers use the same formula. A little farther down, he states that Dr. Royston Riggott's aplanatic searcher is an apparatus for getting a greater amplifying power, when, in fact, its main purpose was to improve the correction of object glasses.

Chapter IV. is common-place, and much behind the times. On page 56, in speaking of Nobert's lines, he reproduces the substance of the paragraph on the same subject in Carpenter's *old* edition of "The Microscope and its Revelations," and he appears never to have heard of the resolution of these lines which within the last few years has become so common. In the next paragraph, he goes on to speak of some of the more common test diatoms, but he is evidently unacquainted with the structure of these objects as seen with modern objectives.

Chapter V., on "The Modern Methods of Examination," being a condensation of chapters seven and eight of Frey's work on the microscope, is better. In many portions, however, the descriptions are so brief as to be almost unintelligible to the beginner, especially when he departs from his master and at-attempts a description on his own responsibility. On page 62, for example, he conveys the impression that substances hardened in watery solutions may be imbedded in wax or paraffin without the withdrawal of the water. The ruin of a few valuable specimens by a trial of this method will be sufficient to convince the novice that there is something wrong in it. Such hasty and imperfect descriptions only do harm, by leading to inevitable disappointment in the novice who, at the best, is doomed to encounter difficulty and disappointment enough to discourage any but the most persevering even when the best is done for him by his author.

Chapter VI., although commonplace enough, is exceedingly incomplete. It is deficient in giving neither the newest methods, nor the best of the old methods. For example, in his description of the method of mounting in balsam, he makes no reference to the method of mounting *wet* preparations by transferring through absolute alcohol to oil of cloves, or turpentine, to balsam; one of the most valuable as well as the most common of the modern methods of mounting. Nor does he speak of the use of balsam in solution, another exceedingly valuable method.

We need hardly take up the time of the reader by entering into examination of the remaining chapters, which are neither better nor worse than those we have gone over. No one of them, we are sure, would be of use as a working manual for any one, either student or expert.

The plates and cuts are nearly all fac similes of well known figures, and are therefore as a rule, good. Still, we hardly think that Dr. Beale would be willing to accept plate V. as a fair representation of his idea of bioplasm, and he certainly would not be willing to admit that the wretched scrawl opposite page 200 was in any sense of the word a copy of the ganglionic nerve cell which he has described. So, too, Fig. 172 Plate XXIII., evidently intended to be a copy of the well known figure in Stricker, illustrating the structure of the infundibula of the lung, entirely misses the point and fails to give the idea intended to be conveyed by the original figure.

We cannot see what is gained by printing the figures in colors. The substance of the lung is not red even in life, neither is the tubule of the kidney. Neither can we see the advantage in printing them in expensive color, and then printing the titles in black, requiring two impressions, unless indeed the object is to make a striking page to catch the eye. Some of these colors positively misrepresent many of the structures which they are intended to show. We would, of course, not include in these remarks such plates as Plate II., which illustrates microscopic sections of minerals, and is a very fair specimen of color printing. nor Plate V., which is intended to illustrate some of Dr. Beale's views, if the execution had only been better. All the others however, in our opinion, would have been better in plain black, and the amount of money which has been used in printing them in colors had been expended in improving their character.

In conclusion, we would say that while the work is not altogether without merit and may be of use to some, more particularly those who desire to get a general idea of the subjects which it treats without engaging in practical work, yet for those who wish a treatise which shall give them help in the practical use of the instrument, we can hardly conceive of a book less useful. L. C.

---

The Theory and Practice of Medicine, By *Frederick T. Roberts, M. D., B. Sc., M. R. C. P.* Second American from the last London edition, revised and enlarged. Philadelphia: *Lindsay & Blakiston.* 1876. Octavo pp. 920.

The first edition of this volume was fully reviewed at the time of its issue in this Journal, so that at this time only a passing notice is necessary. We fully endorse the statement of the reviewer of that edition when he says that "so clear, terse and pointed is the style; so accurate the diction, and so varied the matter of this book, that it is almost a dictionary of practical medicine."

The present edition has been carefully revised, several subjects in great part re-written, and an earnest effort made to bring it up to the times. Among the valuable additions that have been made is the separate chapter on "The Diagnosis of Acute Specific Diseases." In this chapter, after a few remarks respecting febricula, he presents in the form of a table the chief clinical features of the principal fevers prevalent in this country, and the diseases for which they are liable to be mistaken; and lastly briefly notices the more peculiar or less common specific diseases.

The chapter on "Diseases of the Skin" has been re-written and somewhat enlarged, and is a brief but very valuable discussion of the diseases that are likely to be met with in a medical practice.

In conclusion, we commend the book to the young student and busy practitioner as a faithful exposition of modern medical theory and practice. D. R. B.

The Anatomy of the Head with six Lithographic Plates Representing Frozen Sections of the Head. By *Thomas Dwight, M. D.*, Professor of Anatomy at the Medical School of Maine, Instructor in Histology at Harvard University, Surgeon at the Carney Hospital and at the Boston Dispensary. Boston: *H. O. Houghton & Co.* 1876.

This little book of 136 pages is a very creditable work, indeed. Of course, Dr. Dwight could say little that was really new regarding the anatomy of the head, except as to the relative position of the several organs and structures. This he has done in his text somewhat, but in his excellent plates more. In anatomy and histology, pictures are great educators; they are good educators if they are true to nature. This is especially true of regional anatomy, the branch of the subject in which we most need instruction. These pictures of Dr. Dwight will be in some degree a surprise to every anatomist. The relations in every plate are different from those of the ideal picture anyone would draw—all of which proves that such a study of the subject as this book presents, was needed.

Lectures on the Physical Diagnosis of Diseases of the Heart, By *Arthur Earnest Sansom, M. D.*, London, etc. *I & A Churchill*, 1876. 12mo.; pp. 115.

We have read this book with great profit, but very little pleasure. With great profit because it is the work of a master hand, presenting in a strict and succinct style the leading principles of the subject of which it treats. With very little pleasure, however, because of the cheap style in which the publishers have issued it. So miserable indeed is the typography, that few will read it without detriment to their eyes.

The volume opens with a "schema" which is the most concise and accurate summary of symptoms, signs and causes of disease of the heart we have ever seen. This is followed by a detailed description of the symptoms based upon a personal observation of one hundred typical cases of disease of the heart, specifying the number of cases in which each special symptom was present. He concludes this chapter by enunciating the important aphorism "that you have never made a complete examination of any patient, whatever his ailment, unless you have estimated as far as possible the condition of his heart."

The next section is devoted to Inspection, and this he begins with the axiom that "you can never make a satisfactory examination in suspected heart disease, unless your patient be stripped to the waist."

He classifies his observations by inspection under (1) hue of the surface, (2) cardiac dyspnœa, (3) œdema, and (4) pulsation. Under the latter heading he concisely states the fact, "that the area of visible pulsations may, however, be considerably increased; although there be no hypertrophy, in nervous palpi tation, in some cases of anæmia, in Grave's disease, or in chorea, pulsation may be observed over a space the size of the palm of the hand; you will note however, in such cases, the apex beat is not displaced from its normal position, nor is the chest wall bulged outward."

Lectures follow devoted to palpation, percussion, and ascultation. In the last chapter he maintains with ability that the murmurs located at the apex or base in chorea are due to organic lesion of the mitral or aortic valves.

In conclusion we quote from the modest preface of the author that the "lectures were intended for those students who have mastered the rudiments of diagnosis and were qualifying themselves for careful observations in the hospital wards. It has been thought that they might prove useful also to practitioners as presenting the essentials for the clinical recognition of Diseases of the Heart, according to the most modern views of cardiac pathology. The work is in no sense encyclopædic: It is intended to be suggestive, not exhaustive, and is founded as far as possible upon personal observation and experience."

D. R. B.

Cyclopædia of the Practice of Medicine. Edited by *Dr. H. Von Ziemssen.* Vol. V. Diseases of the Respiratory Organs. By *Prof. Juergensen*, of Tuebingen; *Prof. Hertz*, of Amsterdam; *Prof. Ruehle*, of Bonn; and *Prof. Rindfleisch*, of Wurtzburg, etc. *William Wood & Co.*

The authors of this volume and their writings, are so well known to the medical world as to need no comment, and this fact would make an extended review of this volume a work of supererogation.

Prof. Juergensen treats quite fully of croupous and catarrhal pneumonia, of hypostatic processes in the lungs, and of pneumonia from embolisms, its causes, diagnosis and treatment.

Prof. Hertz contributes articles on anæmia, hyperæmia and œdema, hemorrhages, atelectasis, atrophy, and hypertrophy of the lungs.

With regard to the latter, after stating the views of Laennec, Morgagni, Rokitansky, Virchow and others, he says: "We come, therefore, to the conclusion that hypertrophy of the lungs does not exist, and that what has hitherto been considered as such is really some other pathological condition."

Regarding the development of emphysema, the same author avoids controversy by accepting in a measure both of the theories as to respiratory pressure and lesions of nutrition.

Unfortunately he can give us nothing upon which to base a favorable prognosis as to the cure of the disease.

We find exhaustive articles by the same author on gangrene of the lungs, new growths in the lungs and mediastinum, and parasites in the lungs. Prof. Ruehle treats of pulmonary consumption and acute miliary tuberculosis, and sixty pages by

Prof. Rindfleisch are devoted to acute and chronic tuberculosis. This is an interesting article, illustrated by numerous plates. This is one of the best of the volumes of this cyclopædia.

COUGHS, CONSUMPTION, AND DIET IN DISEASE. By *Horace Dobell*, M. D., F. R. M. C. S., etc. *D. C. Brinton.* Philadelphia, 1877.

This little work is made up of a series of extracts from the various published lectures of the author.

It is written in a clear and forcible style, and is founded upon an extensive experience which will render it a valuable acquisition to the literature of these subjects.

After treating of the diagnosis of phthisis, the author discusses the relations existing between bronchitis and emphysema, and details the history of neglected coughs. He dwells at considerable length upon the importance of post-nasal catarrh in the production of winter cough.

After trying a great number of applications in the form of spray, gargle, lotion, inhalation, snuff and lozenge, he has concluded that the best applications for this disease are,

1st. A medicated injection consisting of borax, ʒi.; glycerine of carbolic acid, ℥ii.; bicarbonate of soda, ℥i. to half a pint of warm water.

2d. A medicated snuff consisting of equal parts of camphor, tannic acid, white sugar and high-dried Welsh snuff.

3d. A medicated lozenge consisting of camphor gr. ii.; tannic acid, gr. $\frac{1}{2}$; hydrochlorate of morphia, gr. $\frac{1}{36}$; white sugar, gr. x.; acacia gum, gr. ii.; and,

4th. A rubefacient liniment, to be rubbed behind the ears and on the back of the neck once or twice daily, for which purpose he recommends pure compound camphor liniment.

He devotes considerable space to the discussion of ear cough, an affection usually overlooked by physicians.

The latter part of the book is devoted to the subject of diet for the sick, and although it presents nothing new to those who have made a study of this subject, still it furnishes many suggestions which will be found very serviceable, especially to the younger members of the profession.

A Treatise on the Theory and Practice of Medicine. By John Syer Bristowe, M. D., London; edited by James H. Hutchinson, M. D., Philadelphia. Henry C. Lea, 1876.

A system of medical practice is a necessity for the student, and even for the practitioner, and yet, in these days of division of labor, the physician of extended reading sometimes wonders that one man who can hardly be supposed to be familiar with all the diseases in the nomenclature, should essay to write a book intended to comprehend the anatomy, pathology, etiology, treatment, etc., of all the disorders in the catalogue. And this impression, that the science and practice of medicine have developed beyond the mental reach of any one man, is deepened by the appearance of such encyclopædias, as Reynolds' system of medicine, Ziemssen's work, etc., as well as by a comparison of the very meagre article on, or mere reference to, certain diseases to be found in some of works on practice, with the elaborate monographs on the same subjects, by men familiar from long study and large experience with that of which they treat especially. It was not then with any particular satisfaction that we found on our table the new large octavo bearing the imprint, "*The Theory and Practice of Medicine, Bristowe.*" But, having scanned the volume with more than usual care, we are convinced that the author has accomplished the difficult task of adding another acceptable volume to the list of books on Theory and Practice, in a manner that will reflect credit upon himself, and prove of decided benefit to the profession.

This book has one particular charm and advantage. It is eminently inductive; it seems to have been intended by the writer as an educational book, such as a student needs, and just such as every practitioner who wishes to keep fresh in his mind the principles and practice of medicine, would be glad to have the opportunity to read *through.* The language, though not so graceful and flowing as that, of that modern classic Watson's Practice, is yet terse and clear, without having that measure of conciseness and condensation which continually taxes the attention and penetration of the reader.

The method of description of diseases is pleasing, it is not over methodic—scholastic—but easy and natural; the current of the description not being interrupted by the artificial divisions common to didactic writers, but being from definition to treatment one continuous recital. The treatment with the exception of a few diseases with which, in all probability from want of opportunity of study, the writer is not familiar, seems to us judicious.

In regard to diseases as yet incurable, no long array of remedies are cited, on the contrary, we meet not unfrequently with expressions such as this: "It is not clear that any remedy exerts any, even the slightest, direct influence over the course of this disease." The author has evidently not considered it his province as a teacher to go beyond what is known in the matter of therapeutics. He is disposed, on the contrary, critically to regard the facts bearing upon the curative power of an agent, and candidly to state his convictions thereon. Upon the whole, we know of no work which we could more confidently recommend to the student or the practitioner, intending a review of the field of Theory and Practice, than this book of Dr. Bristowes.

We thus commend it, because the vast array of facts pertaining to the practice of medicine, as it is to-day, are here presented ably, and with that method, order and perspicuity which, in all departments of education, distinguish the lessons of an acceptable and profitable teacher.

Part I. embraces Etiology, Physiological Processes in Health, Physiological Processes in Disease, etc. These chapters contain an exposition of the various subjects fully up to the times; the author writing as one familiar from his own observation, with the more recent and important facts and theories bearing on this subject.

Part II. embraces special pathology, in which the diseases of the various organs are treated of in a systematic manner, and with as much thoroughness as would appear to be consistent with the plan of the work. Upon this section of the book we make the following comments:

The opinion that anasarca occurs more frequently, after mild than severe cases of scarlet fever, is endorsed by the author.

A convalescent from scarlatina should be confined to his room, either in bed or incased in flannels. The treatment by large doses of carbonate of ammonia receives a mere mention.

Opium is recommended for relieving the pains and mitigating the delirium of the stage of invasion of small pox, and especially during the secondary fever. Cow pox is not considered as identical with small-pox. As the discoverers of vaccination, reference is made to the school-master Plett, of Holstein, as having used vaccination in 1771, three years earlier than Benjamin Jesty. The author is of the opinion that scrofula and syphilis are occasionally inoculated by the use of vaccine from the arms of infected children. Specific contagious diseases are believed to depend upon living organism of a fungoid nature.

The resemblance between dengue and relapsing fever is pointed out, and the following query is put, "can the dandy fever be the relapsing fever modified by climate?"

Of epidemic cerebro-spinal meningitis, the author says, there can be little doubt that it is infectious. The chapter on diphtheria is headed "Diphtheria (Membranous Croup)" and he declares the diseases commonly described under these two heads to be one and the same disorder. As to the operation of tracheotony in diphtheritic croup, the author evidently endorses the maxim, "never operate too late; it is never too late to operate."

Typhoid fever is regarded as "*par excellence* the fever of fecal decomposition."

The treatment of cholera, as given by the author, is very unsatisfactory. He does not attach importance to the treatment of the premonitory diarrhœa. He confidently states his belief "that if a case be simple diarrhœa, it will not run into cholera, and if it be one of commencing cholera, there is no more ground for believing that it can be cut short, than that typhoid fever or whooping-cough may be." Will any physician who has passed through severe cholera epidemics, fail to pronounce these statements as false and full of danger?

From the chapter on syphilis, we extract the following sentence: "It is now established beyond doubt, * * * * *

that a person fully under the influence of the syphilitic poison, or who has had an attack from which he has recovered, very rarely, indeed, acquires a chancre, even when inoculated under the most advantageous circumstances, and even more rarely suffers in consequence from the secondary symptoms which so surely follow on the primary inoculation." How the first part of this statement can be made to tally with the fact that inoculations in the method of syphilization have been successfully repeated on the same syphilitic individual, hundreds and thousands of times, may be left to the judgment of the reader.

In the causation and history of ague, it is stated that the disease does not spread from a centre, successively invading town after town, and county after county. This statement is perhaps controvertible, as instances of such invasions are on record. The author denies the influence usually assigned to decaying vegetable matter as a cause of ague, and inclines to refer its origin to contagia similar in nature to those of infectious fever.

In the treatment of malarious fevers, the author seems to have in mind the management of the intermittent type mainly.

The directions, so far as the severe forms of malarial poisoning, remittent and pernicious fevers, are concerned, are entirely too meagre, and therefore defective and insufficient.

In the treatment of erysipelas, the author says: "Ammonia, camphor, iron, quinine, have all been employed in erysipelas, and all are recommended. It is questionable whether any one of them is of material use during the febrile stage of the disease." Certainly this declaration, so far as it refers to iron and quinine, will not receive the general endorsement of American physicians.

We find in the article on percussion this statement, which, we think, will meet the endorsement of many practitioners, and we are the more disposed to quote it, because the race of percussors who claim to be able to detect in the lungs "a tubercle no larger than a grain of wheat" may not yet be extinct. "But although marked dullness is always present when consolidation is extensive, and continuous, it is often absent, or at all events scarcely appreciable, either when an extensive tract of lung-tissue uniformly contains more solid matter or fluid, and less air than natural, or when miliary or larger nodules of solid tissue, separated from one another by a network of crepitant tissue, are even thickly distributed. Thus congested or œdematous lungs, and lungs in the early stage of inflammation, on the one hand, and lungs which are the seat of disseminated tubercles or of lobular pneumonia on the other, are not unfrequently so strikingly resonant as utterly to deceive the too confiding percussor."

Bronchophony and pectoriloquy, are not regarded as mere grades of the same phenomenon. "Bronchophony never becomes converted into pectoriloquy. Bronchophony is the offspring of laryngeal intonation; pectoriloquy of the articulate sounds developed within the cavity of the mouth."

In the treatment of both laryngitis and tonsillitis, the author recommends opium as giving great relief—a recommendation which is here noticed because of our belief, that the misery of these diseases is frequently allowed by practitioners to go unassuaged, when a portion of morphine would greatly mitigate the distress.

In pleurisy, vocal fremitus is said to be suppressed over the dull areas. In the treatment of large pleuritic effusion, the author has little faith in the ordinary remedies, and inclines toward the early use of the trocar, when such interference seems called for.

The author is not disposed to adopt the view of Virchow, as regards tubercle; he is inclined to regard the miliary caseous forms as varieties of the same diseased product. The experiments of Villemin and others apparently showing the inoculability of tubercle; and also that tubercle may be developed by the injection of non-tuberculous matter, are regarded by the author as conclusive.

Our author, remarking upon spasm of the trachea says, how far this condition may be a matter of clinical importance is questionable. Dr. Bristowe believes, however, that when aneurismal or other tumors are compressing the trachea, and inducing from time to time spasmodic attacks of dyspnœa, the immediate cause of the difficulty of breathing is not unfrequently spasmodic contraction of the muscular tissue of the compressed portion of the trachea, associated, it may be, with more or less hyperæmia, and accumulation of mucus.

Observation and study of attacks of dyspnœa occurring in children after tracheotomy for croup, have convinced us, that certain of these attacks, originating apparently from irritation of the trachea in changing the outer tube, while they are clearly spasmodic, are yet not referable to spasm of the trachea, but to spasm of the bronchioles, induced by the irritation of the parent trunk at the site of the wound. A careful study of the curious facts in the literature of tracheotomy having reference to spasmodic dyspnœa occurring during the "after treatment" or incident in some cases to attempts to remove the tube after restoration of the primarily diseased larynx, will, we think, corroborate this idea.

Dr. Bristowe unhesitatingly attributes Addison's Disease to tubercular infiltration of the suprarenal bodies.

Phlegmasia alba dolens, the author writes, "is due to thrombosis of the trunk veins of the limb, or of the larger veins to which these converge." To the reader bearing in mind the words of Fordyce Barker, "the relation which the thrombosis bears to phlegmasia dolens seems to me to be that of an effect rather than a cause," and again, "I believe that the blocking up of the veins by thrombosis is one of the conservative efforts of nature to promote recovery," such a declaration would seem positive beyond the facts.

In reference to delirium tremens, our author after saying, "recent inquiries show that it is the immediate consequence of excessive drinking," adds, "It may, no doubt, supervene at the time when such persons (excessive drinkers) are commencing to abstain, but not in consequence of their abstinence. This doctrine that mania a potu is not caused by abstracting drink from the drunkard has long had supporters in England, but we think few in this country hold it to be true. On the contrary, so far as our experience goes, it is universally admitted that delirium tremens is very often the direct result of withholding the usual supply of liquor. Dr. Bristowe refers to Dr. Locock, Gairdner, Wilks, Anstie and others, as now maintaining that delirium tremens is a disease of low mortality, tending to get well of itself, and that opium is not only not needed, but that its use is attended with no inconsiderable danger. Is not the credit of first publishing these facts due to Dr. John Ware, of Boston? As to the treatment the author is quite unsatisfactory. After some good advice as to the general management of the patient, he continues: "Those who consider sleep indispensable would now administer either chloral or some preparation of opium. The chloral is sometimes given with advantage in doses of from ten to twenty grains every half hour till sleep is induced. Opium or morphia may also be given in comparatively small doses at short intervals. It is better, however, we believe, to administer it from the beginning in large doses, and to repeat it or not, according to its effects; to give for example from half a grain to a grain of morphia, or from a half a drachm to a drachm of laudanum at once, and to repeat the medicine in smaller doses at intervals of an hour or two, if sleep be not induced. It may be well to add that patients with delirium tremens are difficult to bring under the influence of narcotics." We object to these directions, qualified as they are by what precedes in the text, as tending to too free use of the articles mentioned. In some cases the repetition of opium, even in comparatively small doses at short intervals till sleep is induced, which, according to Dr. Ware, might be five or seven days, might not be free from danger. Under the old theory of "sleep or death," we remember one case in which a tablespoonful of laudanum was repeated every half an hour, for some time, without inducing sleep, until suddenly the respirations fell to seven per minute, coma and death speedily following. To the writer who once collected statistics of some 250 cases of delirium tremens treated by "opium and the straight jacket," 33 per cent. of which proved fatal, even the guarded recommendation of the narcotic, here quoted, savors of danger.

In acute gastritis, the author recommends opiates in large doses preferably, subcutaneously.

In noting the several modes of onset in peritonitis, that by intense shock or collapse is omitted; though further on it is said: "Even in the early stage of the disease, when the pulse is little accelerated and sharp, perhaps strong, a little over-exertion, or some unwonted effort may induce dangerous collapse." And again, in speaking of peritonitis, he says: "In the course of the disease, extreme and immediate collapse may occur, so that the symptoms may simulate those of fatal intestinal hemorrhage, or of rupture of an aneurism, or of the heart, or of cholera, fatal without emesis or purgation." In referring to the results of an attack of peritonitis, the author remarks: "In some cases, slowly contracting adhesions compress a length of bowel, and render it practically impervious." In speaking of the pathology of rheumatism, Dr. Bristowe asks: "Is it a local disease, or a constitutional disease?" and in answer to the query he says: "It seems to us that there is little or nothing in rheumatism in respect of its proximate cause, to distinguish it from pneumonia, bronchitis, nephritis, erysipelas, or other examples of local inflammations caused by exposure to cold, or cognate conditions.

In regard to tape-worm, the author repeats the statement generally found in the book, that the tænia solium is one of the most common of human tape-worms, remarking, however, that the tænia mediocanellata which was formerly confounded with T. solium, is equally common. In the observation of the writer, only one case of tænia solium has been noted in a series of 40 cases, of which 38 were T. mediocannellatæ. The author declares that the symptoms produced by tape-worm, are, upon the whole, trivial and unimportant, many of those infected, enjoying perfectly good health. This is a statement much needed, though it should have been added that the direst symptoms, attributed to tape-worm, occasionally actually depend upon it. No reference is made to an increasing frequency in the occurrence of these parasites, whereas in this city it is probable that patients suffering from tape-worm are met with ten times as frequently as formerly. In treating of thread-worms, the author says, local measures are usually amply sufficient for the riddance, such as strong infusion of green tea, etc., this statement contrasts strongly with that of practitioners, who declare the eradication of these pests next to impossible. J. B.

### BOOKS AND PAMPHLETS RECEIVED.

On the Nomenclature and Classification of Diseases of the Skin. By L. Duncan Bulkley, A. M., M. D.

Restriction and Prevention of Scarlet Fever. Document issued by the State Board of Health of Michigan.

Demilt Dispensary Department of Diseases of the Skin.

Classification of Diseases of the Skin.

Tracheotomy in Diphtheria. By J. H. Pooley, M. D.

The Woman's Hospital in 1874. A reply to the printed circular of Drs. E. R. Peaslee, T. A. Emmet, and T. Gaillard Thomas, addressed "To The Medical Profession," May 5th. By J. Marion Sims, M. D.

The Woodruff Scientific Expedition Around the World. 1877–79.

Report of the Board of Health of Quincy, Illinois. For the year ending March 31, 1877.

Twenty-Eighth Annual Announcement of the Woman's Medical College of Pennsylvania, Phila., 1877-78

Review of Dr. Squibb's Proposed Plan for the Future Revision of the U. S. Pharmacopœia, being a special report upon this subject. By the Committee of National College of Pharmacy, Washington, D. C., at a special meeting, held May, 28, 1877.

Titles of the Acts of the Thirteenth General Assembly of the State of Illinois, Approved or Vetoed by the Governor. Also a list of the Members of State Educational, Charitable and Penal Institutions, and the Canal and Railroad and Warehouse Commissioners.

The Model Physician and Model Patient. A Valedictory Address Delivered in Wieting Hall, Syracuse, Feb. 19th, 1875. By H. D. Didama, M. D.

Transactions of the Pathological Society of Philadelphia, for 1875--6.

An Elementary Treatise on Practical Chemistry and Quantitative Inorganic Analysis, etc. By Frank Clowes, D. Sc., etc., etc. Philadelphia. H. C. Lea. 1877.

Transactions of the American Gynecological Society. Vol. I. For the year 1876.

Guy's Hospital Reports. Series III. Vol. XXII, 1877.

Disease of the Mind. By Chas. F. Folsom, M. D.

Medical and Surgical Reports of the Boston City Hospital.

Surgical Observations with Cases and Operations. By J. Mason Warren, M. D., 1877.

Ziemssen's Cyclopædia of the Practice of Medicine. Vol. XII. Diseases of the Nervous System. Vol. XV. Diseases of the Kidney.

Notes on the Epidemiology of Ohio. By Thomas C. Minor, M. D.

Scarlatina Statistics of the United States. By Thomas C. Minor, M. D.

The Association of American Medical Colleges. History of its Organization, its Constitution, By-laws, Articles on Confederation, and List of Members. 1877.

The Specialty of Diseases of Women. By Clifton E. Wing, M. D.

Syphilitic Phthisis. A paper read before the Missouri State Medical Association. By Wm. Porter, M. D.

A Case of Abdominal Pregnancy Treated by Laparotomy. By T. Gaillard Thomas, M. D.

The Prophylactic Treatment of Placenta Prævia. By T. Gaillard Thomas, M. D.

Pus in Ovarian Fluids. By James R. Chadwick, M. D.

Alcohol as a Food and Medicine. A Paper from the Transactions of the International Medical Congress. By Ezra M. Hunt, M. D.

On the Diagnosis of Urethral Stricture by Bulbous Bougies, with Illustrative Cases. By J. William White, M. D.

Report of Management of the Insane, of Great Britain. By H. B. Wilbur, M. D.

Viburnum Prunifolium (Black Haw). Its Use in the Treatment in the Diseases of Women. By Edward W. Jenks, M. D.

Calculi Found in the Bladder after the Cure of Vesico-Vaginal Fistula. By Henry F. Campbell, M. D.

History of a Case of Recurring Sarcomatous Tumor of the Orbit in a Child. Illustrated by Thomas Hay, M. D.

Practical Points in the Electrolytic Treatment of Cystic and Fibroid Tumors. By George M. Beard, M. D.

Pneumatic Selef—Replacement of the Gravid and Non-Gravid Uterus. By H. F. Campbell, M. D.

Cholera: The Laws of its Occurrence, Non-occurrence, and its Nature. By C. Sprinzig, M. D.

Note on Inherited Effects of Lesions of the Sympathetic Nerve and Corpora Restiformia on the Eye. By Eugene Dupuy.

Transactions of the Nebraska State Medical Society, at its Sixth, Seventh and Eighth Annual Meetings.

First Annual Report of the State Board of Health of the State of Wisconsin, 1876.

Thirty-Fifth Annual Announcement of Rush Medical College, Chicago 1877-78.

The Detroit Medical College Announcement and Catalogue, 1877-78.

The Annual Announcement of the Department of Medicine and Surgery of the University of Michigan for 187-778.

Thirteenth Annual Report of the Board of Managers of the Washingtonian Home of Chicago.

On Albumen in the Treatment of Pulmonary Consumption. By E. L. Shurly, M. D., etc. 1877.

What is the Comparative Physiological and Therapeutic Action of Free Phosphorus and the Hypophosphites? An Essay to which the "Merrit H. Caste" Prize was awarded by the New York State Medical Society. By Samuel P. Percy, M. D.

Rare Forms of Umbilical Hernia in the Fœtus. By James R. Chadwick, M. D., etc.

The Discovery of Anæsthesia. By J. Marion Sims, M. D., etc.

Labor complicated with Uterine Fibroids and Placenta Previa. By James R. Chadwick, M. D., etc.

Two cases of Morphœa: with remarks on the disease and its differential diagnosis. By L. Duncan Bulkley, M. D.

